# Promising Practices to Promote Snap at Schools: A Quantitative Analysis of Snap Outreach Efforts

**DOI:** 10.1111/josh.70255

**Published:** 2026-10-04

**Authors:** Jennifer Salt Taylor, Kiersten Koch, Jenna DeSimone, Ricardo Kairios, Veronica Jones

**Affiliations:** ^1^ Department of Family and Community Health Sciences, Rutgers Cooperative Extension, School of Environmental and Biological Sciences Rutgers, The State University of New Jersey New Brunswick New Jersey USA

**Keywords:** child health, community partnerships, food insecurity, stigma, Supplemental Nutrition Assistance Program (SNAP)

## Abstract

**Background:**

In 2023, 17.9% of US households with children experienced food insecurity. While the Supplemental Nutrition Assistance Program (SNAP) aims to reduce food insecurity, persistent under‐enrollment, labeled the “SNAP Gap,” limits its impact. Schools represent a strategic yet underutilized setting to reach eligible families. This study examined school‐based strategies to increase caregivers' awareness and knowledge of SNAP.

**Methods:**

Twelve diverse school districts implemented stigma‐free SNAP outreach initiatives, reaching 8601 participants across 37 outreach events statewide. Caregiver knowledge and perceptions about SNAP were then assessed via online retrospective post‐then‐pre surveys and analyzed using descriptive statistics and chi‐square tests.

**Results:**

Among caregivers (*n* = 1144), SNAP outreach was associated with statistically significant increases in knowledge (*p* < 0.001), including awareness of benefits, eligibility, application sites, and available assistance.

**Implications for School Health Policy, Practice, and Equity:**

Tailored, school‐based outreach can reduce stigma, normalize use, and increase knowledge of SNAP. Partnerships with community organizations enhance support while minimizing school staff burden.

**Conclusions:**

Schools are effective access points for narrowing the SNAP Gap through outreach, education, and stigma reduction. Future research should examine student perspectives on SNAP and school meal program participation.

## 1 Introduction

Food insecurity, defined as limited or uncertain access to adequate food, affected 13.5% of US households in 2023, including 17.9% of households with children [1, 2, 3, 4, 5]. As food insecurity increases, individuals may experience poorer diet quality, reduced food intake, and disrupted eating patterns [1, 2, 4, 5]. Children are particularly vulnerable, with food insecurity associated with adverse physical, developmental, academic, behavioral, and mental health outcomes [3, 4, 6, 7].

To address food insecurity, the US Department of Agriculture administers the Supplemental Nutrition Assistance Program (SNAP), the nation's largest nutrition assistance program [8, 9]. In 2024, SNAP provided more than $114 billion in benefits to approximately 44 million individuals, improving food security while generating economic benefits for local communities [10, 11, 12]. Despite the availability of application assistance programs, a gap remains between SNAP eligibility and participation, often referred to as the “SNAP Gap” [13]. Nationally, approximately 12% of eligible individuals did not participate in SNAP in 2022, with particularly low participation among the working poor [11, 14]. In New Jersey, closing this participation gap could generate an estimated $239 million in additional economic activity annually [12, 13]. These findings highlight the need for strategies that increase awareness of and access to SNAP among eligible households.

Schools serve as vital community hubs, connecting children and families to vital resources, support, and guidance beyond traditional academics [15, 16, 17]. They are often considered trusted environments where children, families, and staff regularly interact with each other, making schools an ideal setting for targeted outreach projects [15, 17]. Because families are already engaged with school activities, initiatives integrated into existing programs can reach a broader audience, increase participation, and build trust in programs that might otherwise feel uncomfortable, unknown, or stigmatized [15, 17]. Additionally, schools provide valuable infrastructure and staff support, often at little to no additional cost, which enhances the feasibility of such programs [16, 17]. Many school‐based initiatives have been successful in promoting stigma‐free practices and reducing logistical barriers (i.e., time, transportation, and cost) by integrating outreach and enrollment efforts into existing school activities [16, 17, 18].

The role of schools has evolved beyond merely education to becoming key partners in advancing public health initiatives, including improving food security and nutrition [17, 18]. Approximately 90% of public schools nationwide participate in the National School Lunch Program (NSLP) and School Breakfast Program. The NSLP is the second largest United States Department of Agriculture (USDA) Food and Nutrition Service (FNS) nutrition assistance program and offers free and reduced‐price lunches to eligible school‐aged children to promote healthy dietary patterns and support both physical health and academic achievement [8]. While there are additional criteria, both SNAP and NSLP use 185% of the Federal Poverty Level to determine income eligibility for the programs in New Jersey. Oftentimes, families face disproportionate challenges accessing consistent nutritional resources; however, schools are uniquely positioned to help bridge this gap [5, 18]. Through programs like the NSLP and by facilitating access to SNAP, schools can serve as vital connectors between families and essential nutrition resources [18]. Leveraging school partnerships for these efforts can help ensure that children and families receive the necessary support in a trusted, accessible environment [15, 16, 17, 18].

Given the similar income eligibility thresholds for SNAP and NSLP, examining SNAP participation among families enrolled in the NSLP provides a critical opportunity to identify facilitators and barriers to program uptake. Prior research suggests that many families who qualify for both programs participate in school meals but not SNAP, pointing to structural, informational, and sociocultural obstacles that limit enrollment [5, 8, 9]. Rutgers University, in collaboration with the New Jersey Office of Food Security Advocate, initiated the SNAP Gap Project, an exploratory, multi‐year study aimed at understanding barriers and motivators influencing SNAP participation among families whose children participate in the NSLP. The project examined the intersection of school meals and SNAP by centering the perspectives of individuals with lived experience through caregiver focus groups and interviews with school staff and county SNAP administrators.

The overarching goal of the SNAP Gap Project was to better understand why eligible families remain unenrolled in SNAP. A secondary objective focused on identifying school‐based strategies to reduce stigma and improve communication about SNAP eligibility and benefits to develop actionable recommendations for increasing awareness, enrollment, and dignity‐centered engagement through schools. This manuscript addresses the secondary objective by presenting findings from year two of the project, which evaluated caregiver knowledge and perceptions of SNAP using post‐then‐pre surveys. By documenting caregiver experiences and identifying gaps in understanding, this research contributes evidence to inform school‐based outreach strategies and policy approaches designed to more effectively connect eligible families to comprehensive nutrition assistance programs.

## 2 Methods

This quantitative analysis focused on SNAP knowledge among caregivers with children in participating schools. The study was led by a team of researchers at Rutgers University and funded through the New Jersey Office of Food Security Advocate. The larger, mixed‐methods study was conducted during the 2022–2023 (Year 1) and 2023–2024 (Year 2) academic years. Year 1 of the study was a pilot with 6 school districts in New Jersey. Building on lessons learned, Year 2 of the study expanded to an additional 6 districts. This manuscript focuses on the data collected during Year 2 of the study.

### 2.1 Participants

Quantitative analysis was conducted through a survey of 1144 caregivers from 12 school districts participating in the project. Participating schools served students across the Pre‐K through 12th‐grade continuum. There were no grade‐level restrictions imposed for participation in project activities. Most respondents (*n* = 1078) identified as parents. Among those who reported gender (*n* = 879), the majority identified as female (87.6%), with fewer identifying as male (11.6%), other gender (0.1%), or preferring not to disclose (0.7%). Reported ages (*n* = 878) spanned a wide range, with nearly half of respondents between 35 and 44 years of age (47.2%), followed by those aged 25–34 years (26.4%) and 45–54 years (18.7%). More caregiver characteristics can be found in Table 1. While the sample reflects primarily parent‐identified, predominantly female caregivers, school recruitment efforts targeted districts with diverse geographic, demographic, and socioeconomic characteristics to ensure a broad representation of school contexts. Figure 1 and Table 2 provide demographic characteristics of enrolled students and NSLP participation rates by district, respectively.

**TABLE 1:** Caregiver survey respondent characteristics.

Characteristics	*n* (%)
*Caregiver role*	*N* = 1144
Parent	1078
Foster parent	4
Guardian	30
Grandparent	8
Other	46
*Gender*	*N* = 879
Female	770 (87.6)
Male	102 (11.6)
Other	1 (0.1)
Prefer not to say	6 (0.7)
*Age (years)*	N = 878
18–24	25 (2.9)
25–34	232 (26.4)
35–44	414 (47.2)
45–54	164 (18.7)
55–64	37 (4.2)
65 or older	6 (0.7)
*Attended promotional event*	N = 907
Yes	259
No	648

*Note:* Participants were permitted to select multiple roles; therefore, percentages may exceed 100%.

**FIGURE 1:**
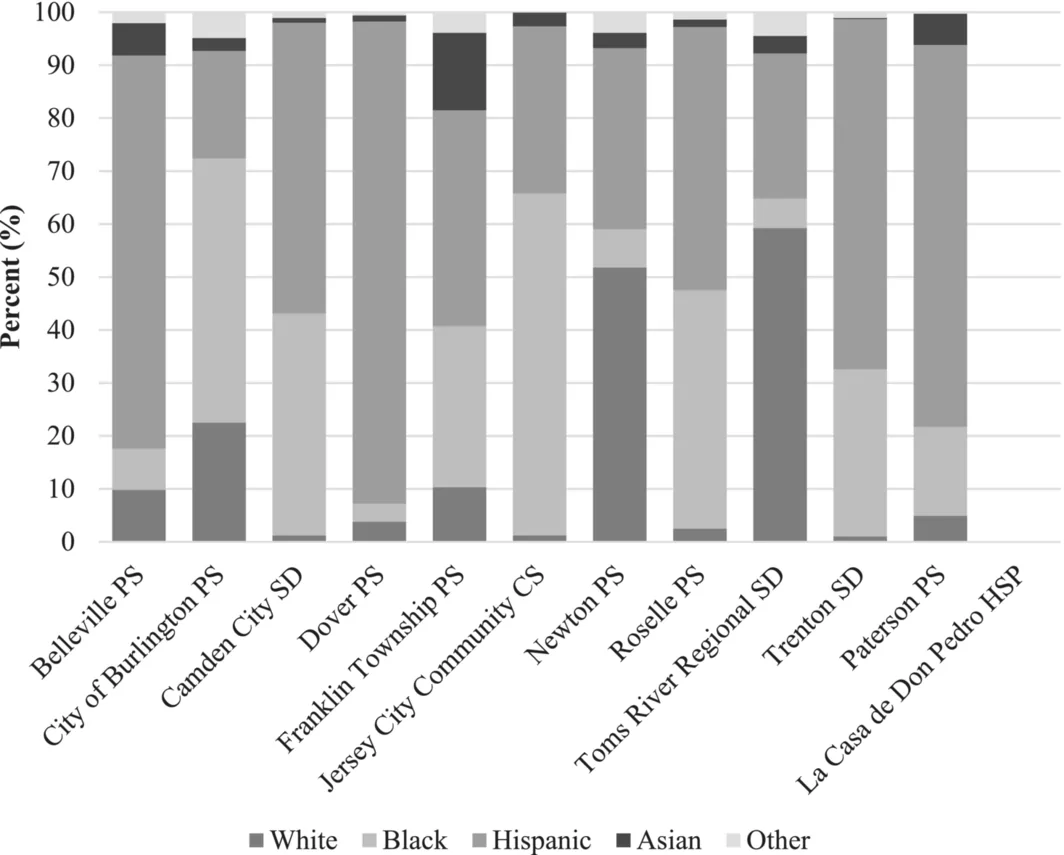
Student demographics by school district. *Note*: La Casa de Don Pedro HSP does not have publicly available data for student demographics. CS = charter school, HSP = Head Start Program, PS = public schools, PSD = public school district, SD = school district.

**TABLE 2:** Enrollment and socioeconomic demographics by school district.

District	Total enrollment, *N*	Free/reduced lunch, *n* (%)	ISP(%)
Belleville PS	5047	3163 (62.7)	46.83
City of Burlington PS	1821	1169 (64.2)	50.14
Camden City SD	6956	3034 (43.6)	77.23
Dover PS	3484.5	1888.5 (54.2)	63.4
Franklin Township PS	7228.5	3263 (45.1)	67.35
Jersey City Community CS	571	428 (75.0)	68.65
Newton PS	1593.5	574 (36.0)	35.3
Roselle PS	3030	1783 (58.8)	45.12
Toms River Regional SD	14289.5	5320.5 (37.2)	33.63
Trenton SD	14,935	6406 (42.9)	64.48
Paterson PS	24,090	14,932 (62.0)	65.25
La Casa de Don Pedro HSP	343	343 (100.0)	—

*Note:* La Casa de Don Pedro HSP does not have publicly available data for ISP.

Abbreviations: CS = charter school; HSP = Head Start Program; ISP = identified student percentage; PS = public schools; PSD = public school district; SD = school district.

### 2.2 Instrumentation

An online survey was developed using Qualtrics Survey Software that included a mix of 5‐point Likert‐scale items, categorical questions, open‐ended responses, and basic demographic questions [19]. The caregiver survey instrument was informed by findings from focus group discussions with caregivers during the pilot year of the study to ensure relevance and clarity. SNAP knowledge was assessed using several items that captured respondents' awareness of SNAP benefits, eligibility, and enrollment processes. Participants were asked whether they had ever heard of SNAP, whether they or someone in their household had ever received benefits, and how familiar they were with the program. Additional items explored common areas of confusion identified during caregiver focus groups during the pilot year, including how respondents learned about SNAP, awareness of minimum monthly benefit amounts, and knowledge of where to apply and get support with the application. The tool was subsequently reviewed by an Associate Extension Specialist in the Department of Human Ecology to assess content and face validity, as well as alignment with study objectives. Because the survey instrument was intended as a community‐based needs assessment, no additional tests of construct validity or reliability were conducted. The survey was designed as a retrospective post‐then‐pre design to reduce response shift bias and survey burden on the participants. The Hunger Vital Sign two question tool was included in the survey to screen for food security [20].

The survey, distributed in English and Spanish, included questions on SNAP familiarity, comfort applying for SNAP at school, participation in promotional activities, and household food security. Participation was voluntary and informed consent was obtained online. No personal identifiable information was collected. Participants were not compensated but could enter a drawing for a $100 electronic gift card. A total of 182 gift cards were distributed to caregivers through a random drawing.

### 2.3 Procedures

Twelve participating school districts implemented school‐based SNAP promotion strategies and distributed caregiver surveys. Districts varied in enrollment size (343 to > 24,000 students), racial and ethnic composition, free and reduced‐price lunch participation (36%–100%), and Identified Student Percentage (ISP). Each participating school district was awarded $25,000 to implement SNAP promotion activities. Schools received 90% of the funding upfront and the remaining 10% was paid upon completion. Schools were encouraged to select outreach methods tailored to their respective communities. Participating schools designated project coordinators to serve as liaisons with the research team. Each coordinator received unique survey links for their district and recruitment materials to distribute to caregivers via email, text, and internal communication platforms. Caregiver surveys were distributed after all promotional activities were completed.

Schools conducted 37 SNAP promotional events that reached 8601 attendees; a detailed breakdown is provided in Table 3. Events included family nights, resource fairs, and community celebrations and ranged from 1 to 6 h in duration. Most events (*n* = 30) occurred during May and June 2024. SNAP information was delivered through promotional materials (70% of events), SNAP Navigators (24%), and SNAP‐Ed staff (5%). Schools used state‐developed and locally created outreach materials and integrated SNAP promotion into existing school and community events to increase reach and reduce stigma associated with program participation. Several districts implemented additional family engagement activities. These included student‐produced bilingual SNAP promotional videos, a district‐wide community cookbook that incorporated SNAP enrollment and food resource information, a SNAP knowledge quiz conducted at school events, and targeted outreach to high school seniors regarding SNAP eligibility during college enrollment. These activities were designed to increase awareness of SNAP and facilitate access to nutrition assistance resources among students and families.

**TABLE 3:** School event distribution by event category and promotional method.

	Promotionalmaterials	SNAPnavigators	SNAP‐Ed	Total
Event type	Number of events (number of participants)			
Community and family: resource fairs and family‐focused events	4 (648)	3 (415)	0 (0)	7 (1063)
Cultural and celebratory: parent meetings and cultural celebrations	11 (1209)	0 (0)	0 (0)	11 (1209)
Education and enrichment: school program registrations, convocations, and skill development events	3 (2250)	4 (2300)	0 (0)	7 (4550)
Health and wellness: health fairs, open gym events, family wellness events, and health awareness events	3 (475)	1 (125)	1 (200)	5 (800)
Student recognition: awards nights, end of year celebrations, and grade specific events	5 (574)	1 (105)	1 (300)	7 (979)
Total	26 (5156)	9 (2945)	2 (500)	37 (8601)

### 2.4 Data Analysis

Survey data were analyzed using SAS Studio software [21]. Respondents with missing key variables (i.e., caregiver type, pre‐ and post‐then‐pretest variables) or who were under the age of 18 were excluded from the analysis (*n* = 141). Descriptive statistics were implemented to provide an overview of findings. A chi‐square analysis was used to examine associations between pre‐ and post‐intervention knowledge levels based on the retrospective post‐then‐pre survey. Event effect was analyzed using Cochran–Mantel–Haenszel statistics to see if attending promotional events had a significant difference. All statistical tests were conducted at a significance level of 0.05.

## 3 Results

To assess changes in knowledge and awareness of SNAP among caregivers following promotional projects at schools, a retrospective post‐then‐pre survey was conducted. Results were collected from over half of the schools (*n* = 7), with all schools invited to participate. The results, *n* = 1144, were then analyzed by school and overall; however, due to a small sample size within individual schools, only data from the overall analysis will be shared.

A chi‐square analysis was used to examine the results of the retrospective post‐then‐pre‐survey for multiple variables. Across all variables, including SNAP benefits, eligibility, where to apply, and where to find help applying, there were statistically significant differences between pre‐ and post‐survey knowledge distributions. For SNAP benefits, knowledge increased from pre to post ($\chi^{2}=32.43,\mathrm{df}=6,p<0.001$). Similar patterns were observed for SNAP eligibility ($\chi^{2}=26.30,\mathrm{df}=6,p<0.001$), where to apply for SNAP ($\chi^{2}=19.96,\mathrm{df}=6,p<0.01$), and where to get help applying ($\chi^{2}=16.71,\mathrm{df}=6,\;p<0.05$). As shown in Table 4, the proportion of respondents reporting moderate or high knowledge increased significantly across all domains, indicating meaningful improvements after interventions were implemented.

**TABLE 4:** Retrospective post‐then‐pre survey: SNAP knowledge and access.

	Pre *n* (%)		Post *n* (%)		Pre vs. post $\chi^{2}$ (df)		
Variable	Event = 1	Event = 0	Event = 1	Event = 0	Event = 1	Event = 0	Event effect $\chi^{2}$ (df)
*SNAP benefits (N = 842)*							
None	50 (20.49)	212 (35.45)	29 (11.89)	136 (22.74)	32.43 (6)***	96.88 (6)***	283.83 (3)***
Low	59 (24.18)	139 (23.24)	41 (16.80)	129 (21.57)			
Moderate	87 (35.66)	180 (30.10)	106 (43.44)	218 (36.45)			
High	48 (19.67)	67 (11.20)	68 (27.87)	115 (19.23)			
*SNAP eligibility (N = 808)*							
None	37 (15.81)	190 (33.10)	22 (9.40)	141 (24.56)	26.30 (6)***	70.93 (6)***	345.17 (3)***
Low	58 (24.79)	152 (26.48)	45 (19.23)	134 (23.34)			
Moderate	84 (35.90)	162 (28.22)	93 (39.74)	201 (35.02)			
High	55 (23.50)	70 (12.20)	74 (31.62)	98 (17.07)			
*Where to apply for SNAP (N = 805)*							
None	28 (12.12)	178 (31.01)	19 (8.23)	135 (23.52)	19.96 (6)**	49.87 (6)***	426.23 (3)***
Low	39 (16.88)	89 (15.51)	30 (12.99)	89 (15.51)			
Moderate	90 (38.96)	186 (32.40)	99 (42.86)	194 (33.80)			
High	74 (32.03)	121 (21.08)	83 (35.93)	156 (27.18)			
*Where to get help applying for SNAP (N = 805)*							
None	30 (12.93)	189 (32.98)	22 (9.48)	135 (23.56)	16.71 (6)*	84.93 (6)***	394.78 (3)***
Low	40 (17.24)	112 (19.55)	30 (12.93)	97 (16.93)			
Moderate	94 (40.52)	154 (26.88)	98 (42.24)	186 (32.46)			
High	58 (29.31)	118 (20.59)	82 (35.34)	155 (27.05)			

*Note:* Percentages reflect only participants with non‐missing data; pre versus post chi square and degrees of freedom reflects results of chi square test comparing the post‐test distribution to the corresponding pretest distribution. Event effect *χ* ^*2*^ values reflect the Cochran–Mantel–Haenszel test evaluating whether the pre‐ to post‐survey change differed between participants who attended the promotional event and those who did not.

Abbreviation: *N* = number of participants with complete data.

* *p* < 0.05.

** *p* < 0.01.

*** *p* < 0.001.

Survey responses also provided insights into caregiver overall familiarity and awareness of SNAP. Over half (54%) of caregivers reported being moderately or very familiar with SNAP, with schools being the primary source of SNAP information. Among caregivers who attended school events, 86% collected SNAP materials and 81% engaged in direct conversations about the program. Additionally, 78% of all caregivers stated they would be interested in learning more. A majority of caregivers (61%) plan to apply for SNAP and 69% indicated they would be comfortable applying at their child's school. Two thirds, 66%, of responding caregivers were not aware of the March 2023 increase of the monthly minimum SNAP benefit in New Jersey to $95.00, highlighting gaps in awareness surrounding recent policy changes.

To evaluate whether knowledge increases varied by event attendance, Cochran–Mantel–Haenszel chi‐square tests were conducted. As demonstrated in Table 4, event attendance was significantly associated with the magnitude of pre‐ to post‐survey change across all variables, including SNAP benefits (CMH $\chi^{2}=283.83,\mathrm{df}=3,p<0.001$), eligibility (CMH $\chi^{2}=345.17,\mathrm{df}=3,p<0.001$), where to apply (CMH $\chi^{2}=426.23,\mathrm{df}=3,p<0.001$), and where to get help applying (CMH $\chi^{2}=394.78,\mathrm{df}=3,p<0.001$). Caregivers who attended the events showed a greater increase from “none” or “low” knowledge toward “moderate” or “high” compared to those who did not attend the event. This suggests that event participation strengthened the impact of the program, contributing to significantly increased knowledge gains.

## 4 Discussion

While SNAP provides vital support in helping families access healthy food, many eligible individuals do not apply for the program. Research confirms that SNAP effectively reduces poverty and improves food security; however, it also reveals barriers to participation and effective implementation [11, 22, 23, 24, 25, 26, 27]. Key obstacles include a complex application process, inadequate benefits, limited access to healthy foods, and stigma [22, 23, 24, 25, 26, 27]. According to the USDA Food Stamp Program Access Study, 45% of eligible but not participating individuals reported stigma as a deterrent to applying for SNAP [24]. Additionally, anticipated stigma can deter SNAP participation, especially among immigrant and marginalized populations [24, 25].

Across all four SNAP knowledge domains, the retrospective post‐then‐pre survey results demonstrated statistically significant improvements in caregivers' understanding of SNAP benefits, eligibility, and application processes. These findings suggest that the outreach efforts implemented throughout this project were effective in increasing overall knowledge. Importantly, the Cochran–Mantel–Haenszel analysis showed that caregivers who attended school‐based events experienced significantly greater knowledge increases compared to those who did not attend. This indicates that school‐based events enhance the impact of the program across the whole school community, with the strongest gains observed among caregivers who attend the events.

Over three quarters of participants expressed interest in receiving more detailed information about SNAP, confirming a clear need for targeted outreach and educational initiatives. The temporary federal increases to SNAP benefits implemented during the COVID‐19 pandemic concluded in February 2022. In response to the resulting reduction in support for New Jersey households, the state initiated a policy raising the minimum monthly SNAP benefit to $95 beginning on March 1, 2023 [28]. Two thirds of survey respondents reported being unaware of this state level enhancement and these findings further highlight a critical need for expanded outreach and communication efforts to ensure eligible residents are informed about available SNAP resources and recent program changes. Outreach will become increasingly important for vulnerable communities due to the planned federal changes to SNAP in the upcoming years. Survey results also highlighted the importance of community engagement in promoting food security resources, aligning with previous research that there are barriers to accurate and reliable information about SNAP [24]. Overcoming these will increase access to the program and build trust within communities.

Across New Jersey, researchers looked at the potential of schools as resources to overcome the barriers discussed. Schools are central to local communities and often serve as a hub, enhancing partnerships, engaging multiple generations of families, and disseminating information to those that may otherwise be unreachable [17]. The variety of schools that participated provided a broad view of school contexts across New Jersey, contributing to a more comprehensive understanding of SNAP‐related outreach in diverse educational settings. Schools can provide space and opportunity to discuss community‐wide issues with multiple generations, create communities of shared interests, and strengthen their partnerships with families.

Schools participating in this project employed a community‐engaged model to proactively share clear, accurate, and accessible information about SNAP and its benefits, primarily through community events, promotional materials, and digital communications. This is consistent with previous research demonstrating that a partnership‐centered model can increase social capital of the community by providing access to a host of holistic services for families [16]. Promoting SNAP at schools through trusted partners helped to dismantle barriers to accessing assistance and ensured families were fully and accurately informed about available resources.

### 4.1 Implications for School Health Policy, Practice, and Equity

Schools can help improve food security and the health of their students by promoting SNAP to families with accurate information about the program [17]. Acting as a community hub, schools increase access to ideas, information, and resources that may be out of reach for many families individually, which will be especially important with the upcoming changes to federal programs like SNAP [15, 16, 17]. Likewise, caregivers who participated in this study identified the potential for schools to serve as trusted hubs for information dissemination and application support.

The statistically significant event effects observed in this study highlight the role of school‐based engagement strategies. Caregivers who attended school events demonstrated larger shifts from low to moderate or high SNAP knowledge, indicating that interactive, in‐person outreach may help reduce confusion, address stigma, as well as provide clear and trusted support for SNAP. These findings emphasize the importance of the school‐based outreach used in this project and may help to explain why community engaged strategies in participating schools were effective. Successful strategies rely on engaging trusted community partners, like SNAP Navigators or administrators at the local level, for real‐time assistance. This relationship supports families without adding burden to school staff's daily responsibilities. These findings align with previous research highlighting the role of school‐community collaboration in effective interventions.

Community‐based initiatives are more effective when stakeholder input drives the framework of the intervention, particularly in regard to cultural relevance and sensitivity [15, 18]. This project allowed schools to decide what activities were most impactful for their communities, as a “one‐size‐fits‐all” approach would not be effective due to the varied demographics of each individual district. For example, some districts hosted cultural events which attracted a wide variety of participants from cultural minorities, many of whom are already disproportionately affected by barriers to SNAP enrollment. However, hosting the same cultural event at a different district that does not have the same demographics may not evoke the same response and may prove ineffective in removing barriers to enrollment.

Participating families prioritized privacy when discussing needs and schools overcame this concern by normalizing the discussion with all community members. Promoting SNAP in stigma‐free and creative ways fosters a collaborative approach to building relationships that increase knowledge about SNAP. Proactive schools have the potential to increase awareness of nutrition assistance programs by creating a supportive environment that reduces stigma and disseminates accurate information through trusted partners, which is consistent with prior literature [17, 18]. This approach further aligns with a previous intervention that aimed to provide community‐based health education services while engaging caregivers in “non‐stigmatizing ways” [18]. This initiative was implemented at the child's school during drop‐off time, ultimately eliminating additional transportation and time barriers that often require caregivers to find alternative opportunities to attend outreach events. Schools enrolled in this study implemented a similar approach by integrating SNAP education into programs that families were likely to attend, thereby removing potential barriers to accessing program information and participation.

Finally, promoting participation in SNAP at schools can generate greater economic benefits for the whole community [11, 12]. As more households access the benefits, the dollars are spent at local food stores, which often employ individuals connected to the school district, such as the caregivers of enrolled students. Financial stimulation at the local level through increased SNAP participation can enhance household stability and foster more secure living conditions for families within the school community.

### 4.2 Limitations

Several limitations of this study warrant consideration. The onboarding of schools occurred at various points throughout the academic year, resulting in unequal durations for program implementation, which may have influenced engagement levels. Additionally, the survey distribution was delayed and ultimately launched during the summer months, which likely contributed to a lower than expected response rate. Notably, some schools yielded no survey responses. Although the retrospective post‐then‐pre survey design may reduce response shift bias, it remains susceptible to recall bias [29]. Because the caregiver survey was developed for community needs assessment and did not undergo formal validity testing, generalizability of the findings may be limited. To address these challenges, adjustments to both the survey format and administration timeline are being refined as this is an ongoing project.

## 5 Conclusions

The study's findings emphasize the role schools play in bridging the SNAP Gap by acting as community hubs for outreach, education, and stigma reduction. By implementing practices that highlight communication and community partnerships, schools offer an opportunity to effectively promote nutrition assistance programs. By exploring caregivers' unique experiences with SNAP, the findings further revealed key barriers to SNAP enrollment, including misinformation, stigma, and limited accessibility. After identifying key barriers, the study explored strategies and practices that could be used to overcome these challenges.

To address food insecurity, schools have the ability to organize initiatives that connect families to various resources, including SNAP. Continued support, staff training, multilingual staff, and streamlined applications are still needed to reduce the SNAP Gap and increase access to benefits. Future studies should examine caregiver and student input to provide further insights into participants' experiences with SNAP and perceptions about nutrition programs. Overall, these school‐based programs offer promising practices to enhance school health and provide a scalable model to promote overall community health.

## Funding

This research was supported by funding from the New Jersey Department of Agriculture and the New Jersey Office of the Food Security Advocate. The authors acknowledge and appreciate their generous support for this work.

## Ethics Statement

This study was approved by Rutgers University Institutional Review Board (Protocol #2023000458). Ethical guidelines were strictly followed, ensuring informed consent, confidentiality, voluntary participation, and minimal risk to participants. Data were securely stored, and all findings were presented in aggregate form to protect participant privacy and maintain the integrity of the study.

## Conflicts of Interest

The authors declare no conflicts of interest.

## Data Availability

The data that support the findings of this study are available from the corresponding author upon reasonable request.
